# Using Music Technology Creatively to Enrich Later-Life: A Literature Review

**DOI:** 10.3389/fpsyg.2019.00117

**Published:** 2019-01-30

**Authors:** Andrea Creech

**Affiliations:** Faculté de Musique, Université Laval, Québec, QC, Canada

**Keywords:** music, technology, creative, aging, later-life

## Abstract

**Background:** A growing body of evidence has demonstrated significant social and emotional benefits of music-making amongst senior citizens. However, several as-yet unresolved age-related barriers to “musicking” have been identified. Positioned within the emergent field of gerontechnology, concerned with the interface between aging and technology research, this review of literature thus explores the potential for music technologies to function as a vehicle for creative musical opportunities in later-life.

**Methods:** ERIC, PsychInfo, and Web of Science databases were searched, focusing on the intersection between music, technology, and aging. The criteria for inclusion were that the paper should: (1) be in English; (2) report empirical research involving the use of music technologies intended to support receptive (listening, interpreting, reflecting) or active (playing, creating, performing) engagement with music amongst older persons, defined as being aged 60 years or above (United Nations, 2017); (3) be published as a peer reviewed journal article.

**Results:** Of 144 papers screened, 18 papers were retained. 10 studies focused on using technology to support musicking in the form of listening, reflecting, and interpreting. Just five studies explored the utility of technology in promoting singing or playing instruments, while a further three were focused on music and movement.

**Conclusions:** Overall, the literature reviewed suggests that older people, even those with complex needs, are capable of, and interested in using music technologies to access and create personally meaningful music. The limited research that does exist points to multiple and significant benefits that may be derived from receptive or active musicking supported by a range of music technologies.

## Introduction

Questions concerned with the potential for music technology to support creative and positive aging are framed within a context where we are witnessing an unprecedented longevity revolution. The world's population aged 60 and above is set to rise from 962 million in 2017 to over two billion in 2050 (United Nations, 2017). Particularly steep increases in absolute numbers of older people will occur in developing countries. All over the world, though, the proportion of the population aged 60 and over is increasing; by the year 2050 some of the most aged countries will be Japan, Spain, Italy, Germany, and Portugal, where those aged 60+ will account for between 38 and 43 per cent of their populations (United Nations, 2013). The year 2050 will see half of the global population living in countries where at least 20 per cent of people are aged 60 years or above (United Nations, 2017). The aging population internationally represents a triumph of public health policy yet poses significant challenges with regards to sustained quality of life, with particular risks of depression and loneliness, chronic disease, cognitive impairments, and sensory declines (Chang-Quan et al., 2010; Sixsmith and Gutman, 2013; World Health Organization, 2015; Chambers et al., 2016)

In response to these quality of life challenges, a growing body of evidence has demonstrated significant social and emotional benefits of music-making amongst senior citizens (Creech et al., 2013a, 2014b; Fung and Lehmberg, 2016). However, several as-yet unresolved age-related barriers to “musicking,” referring to listening, playing, creating, performing, interpreting, and reflecting (Elliott and Silverman, 2015), have been identified (Jansen, 2005; Withnall, 2010; Hallam et al., 2012). These barriers include ageist attitudes, psychosocial factors, as well as issues relating to the social or physical environment, accessibility of conventional tools (e.g., musical instruments), inclusion, and modes of communication. There is now a pressing need to consolidate and develop our knowledge concerning the potential for innovative music technologies to mitigate these barriers, thus maximizing the potential for access to the creative, psychological, and physiological benefits of musical engagement in later-life.

In this paper I therefore review the literature concerned with the utility and efficacy of music technology in later life contexts, addressing the following question: What is the potential for music technologies to function as a vehicle for promoting access to, and engagement in, musicking in later-life?

## Background

### The Power of Music in Later-Life

Listening to music and playing musical instruments have been described by community-dwelling elders as “restorative activities” (Jansen and von Sadovszky, 2004): activities that “enable a person to feel refreshed, rested, at peace, clear-headed, and mentally able to take on new tasks and challenges” (Jansen, 2005, p. 37). By “restorative,” Jansen (2005) refers to activities that enhance well-being through fostering a sense of “being away” (p. 37), either physically in a new location or metaphorically allowing the mind to wander to another place. Restorative activities such as music listening or music-making offer the scope for a person to “feel in a whole other world,” with their interest fully engaged and a high degree of “fit and harmony between the activity and the person's interests, wishes, and abilities” (p. 37).

From a more proactive perspective, it has been argued that creative spaces offer older people the opportunity to protest against a narrative of decline and instead perform creative acts, explore new ways of “becoming” and experience a humanized old age and continuing sense of citizenship. Writing through the lens of very old age, Erikson and Erikson (1998) reflect that the final life stage need not be solely concerned with withdrawal but, rather, may be experienced as a period of growth and creative expression that is accessed through “contact with one another and […] a regaining of lost skills, including play, activity, joy, and song […]” (p. 227).

A body of research supports the view that receptive or active musical engagement may function both as a restorative and as a creative space. (Jenkins, 2011), for example, reported that participation in music was one significant predictor of positive changes in well-being amongst a sample drawn from the English Longitudinal Study of Aging (*n* ≈ 6,000). In a similar vein, Cohen (2006) found that creative musical opportunities were associated with improvements in general health and a reduction in health risk factors. It has been reported that older people who were actively engaged in musical activities felt that they had greater control over their lives, experienced more pleasure and felt more cared for than those participating in other leisure activities; these positive outcomes were found amongst older people who identified themselves as “beginners” in music as well as those who said they were more experienced (Creech et al., 2013b). Creech et al. (2014a) reported that older people formulated aspirations in relation to possible musical selves through playing and performing in musical groups. Reported cognitive advantages include improvements in attention and concentration (Bugos et al., 2007). Describing an ethnographic study in the context of residential care for older persons, Allison (2008) reported that a song writing group provided a space where older people could transcend the limitations of chronic ill health and institutional care, engaging in creative music-making. Thus, the accumulated evidence suggests that engagement with music, including listening as well as making music, offers a context where older people may continue to experience a positive and creative quality of life.

### The Digital Divide

Within our current context, characterized by an aging population alongside the accelerated use of digital technology (Warschauer and Matuchniak, 2010; Poushter, 2016), the field of gerontechnology has emerged, concerned with the scientific study of the interface between aging and technology (Fozard et al., 2000). Increasingly, attention has been directed toward the possibilities offered by technology for enhancing the quality of later life, for example with technological innovations relating to connection with the outside world, ambient-assisted living, e-health, home-monitoring, robotics for independent living, and digital games (Kaplan et al., 2013; Helfert et al., 2015). According to the World Health Organization (2015, p. 36) “emerging technologies, particularly those used to foster communication and engagement,” may make the goal of aging in place “more achievable.”

Although anxieties relating to technology use have been noted amongst older people, Heinz et al. (2013) also found an openness to using technology, particularly innovations that offered potential enhancements to quality of life and independence. This suggests important opportunities exist to explore the potential benefits of integrating technology within the domain of practice of later-life musicking (Stige, 2012; Elliott and Silverman, 2015). Music technology with younger people is now well-established within special education (Farrimond et al., 2011), music education (Ruthmann and Hebert, 2012) and within music therapy contexts (Hahna et al., 2012; Magee, 2013; Stensaeth and Magee, 2016). However, there has been limited attention focused on the use of music technology as a medium to promote access to the holistic and social praxis of musicking (Elliott and Silverman, 2015) in *later life*, particularly in non-therapeutic contexts.

Notwithstanding these technological potentials and some evidence of increasing later-life engagement with technology, a persistent generational digital divide has been noted, whereby older people, in comparison with younger people, remain less likely to engage with technology (Charness and Boot, 2009; Allen, 2013; Poushter, 2016). This so-called digital divide may be related to attitudinal barriers, including those expressed by older people themselves about what it means to be an older person (Jansen, 2005). The generational digital divide has furthermore been linked to obstacles relating to “age-related changes in perceptual, cognitive, and motor systems” (Charness and Boot, p. 255) which may include lack of training; physical challenges when using peripheral equipment such as keyboards; accessibility issues related to tremors, arthritis or limited vision; or cognitive challenges such as difficulty in understanding menu structures.

Amongst music technology users, a digital divide has likewise been observed, contrasting “digital natives” who have grown up with technology with “digital immigrants,” for whom technology represents a foreign and somewhat unwelcoming territory (Magee, 2013). Ambivalence toward music technology, amongst older people, may be related to “an industry-wide focus on youth which has led to a systematic disregard for the needs, preferences, and capabilities of older adults” (Damant and Knapp, 2015, p. 18). Accordingly, technological challenges and accessibility issues may be conceptualized as limits of the technology and its application, not deficits in the users. This approach is derived from the *cognitive system engineering* framework (Hollnagel and Woods, 2005) which takes the perspective that the human agent and the information system form a single unit (the joint cognitive system). Consequently, the purpose of the system—here, music technologies—is to work in conjunction with the human user in an efficient and integrated manner. In other words, one must adapt a system to fit the human capacities and limits. In this way, music technologies may be mobilized and adapted in creative ways that address quality of life issues in later-life contexts.

### Creative Music Technologies in Later Life

According to Himonides and Purves (2010, p 123–124), music technology can “enhance our lives through experiencing music in new ways; facilitate the communication of *our musics* […]; provide wider access to other people's musics […]; and provide access to music for people with special needs and requirements.” Four key terms, drawing upon these principles of inclusion and access to communicative musical experience, frame a design ethos that underpins the development of creative technologies that may be relevant for older people. *Accessible* or *assistive devices* differentiate for specific physical or cognitive constraints, while *inclusive* or *universal* devices are intended to enhance usability through recognition of a wide spectrum of capabilities (Samuels, 2014).

Broadly, creative music technologies comprise electrical or digital tools to select, listen to, create, manipulate, analyse, or record musical sounds. These technologies comprise listening devices, sound generators (e.g., synthesizers or samplers), musical instruments and interfaces (e.g., MIDI controller keyboards; distance sensors; assistive digital instruments) and visual realities (e.g., software that translates musical sound into visual representations and feedback (Farrimond et al., 2011). Sound generators provide a rich musical palette that can be easily accessed and manipulated to meet individual musical preferences. Digital musical interfaces offer the vehicle through which individuals may access and control the sound generators; a range of interfaces are available, and specific interfaces may be chosen for creative reasons or to meet the physical, sensory or cognitive abilities and needs of an individual, while visual realities may be used as motivational feedback tools, and to promote multisensory creative musical expression. Owing to the availability of inexpensive and powerful technology, there is ever-increasing availability of creative music technologies that meet a wide range of diverse needs, including specific needs that may be related to aging.

Cappelen and Anderson (2014, p. 4) have coined the term “Musicking Tangibles” designed to function as an arena for “positive and empowering” musicking experiences within special needs contexts. Arguing that Midi-based assistive music technology, with its emphasis on control of the interface, can be aesthetically limiting and tiring, Cappelen and Anderson advocate a focus on “motivating social interaction, co-creation, and musicking.” Four generations of Musicking Tangible design are described, including multi-sensory and interactive soft objects and spaces that can be “open to many interpretations, interaction forms and activity levels” (p. 5). To date, the reported applications of Musicking Tangibles have focused on families with children with severe disabilities, although the principles of co-creation and empowerment may have significant implications for the design and use of music technology across many life stages and contexts.

Weisberger (2013), who facilitated an elders' song writing group within a Hispanic community adult day center, has described the potential for widely available technologies to support collaborative musicking with older people. Her aim with the group, which included several participants who lived with dementia as well as physical constraints, was to use culturally-relevant music as a unifying medium for the group, many of whose participants had experienced significant isolation related to their cognitive and physical challenges. Early sessions focused around acoustic instruments, which posed many barriers to participation. As Weisberger (2013, p. 284) explains, although the group members could identify the music that “moved” them, “we would lose the beat, get out of tune, forget the words, and get distracted.” GarageBand was adopted as a tool for exploring potential solutions to the limitations experienced with the acoustic instruments, and the group proceeded to create an album of 20 original songs. GarageBand provided a framework within which the group could explore tempo, rhythm, timbre, harmony, and melody. Group members also became confident in using the microphone to experiment and record their singing, learnt new technological language, and contributed to editorial decisions. Weisberger notes that in addition to supporting the exploration of communication and expression through music, GarageBand served as a vehicle for helping to normalize the wider technological world. She further notes that a range of non-directive as well as directive facilitation strategies were necessary in order to support the participants effectively in reaching individual as well as group goals. For example, while some participants strove to enhance memory or language, others used the musical activities as a context for reducing anxiety, and these goals were situated within a group context that prioritized mutual support and reciprocal recognition of individual expressions of feelings or experience. Weisberger captured individual expressive ideas using multiple tracks, loops and layering, achieving a sense of the whole being aesthetically, greater than the sum of its parts. Finally, Weisberger observes that the option of listening back to recorded performance contributed to and sense of pride in the group identity, as well as boosting self-awareness and self-esteem.

The background literature thus suggests that there may be an important role for music technology to play in facilitating access to creative music opportunities for older people, including frail elders who may be living with significant cognitive or physical challenges. However, to date there has been limited empirical research in this area. The purpose of this review was therefore to synthesize the previous research concerned with music technology in later life, with a view to identifying key messages and themes, as well as areas for future exploration.

## Methods

**Figure 1:**
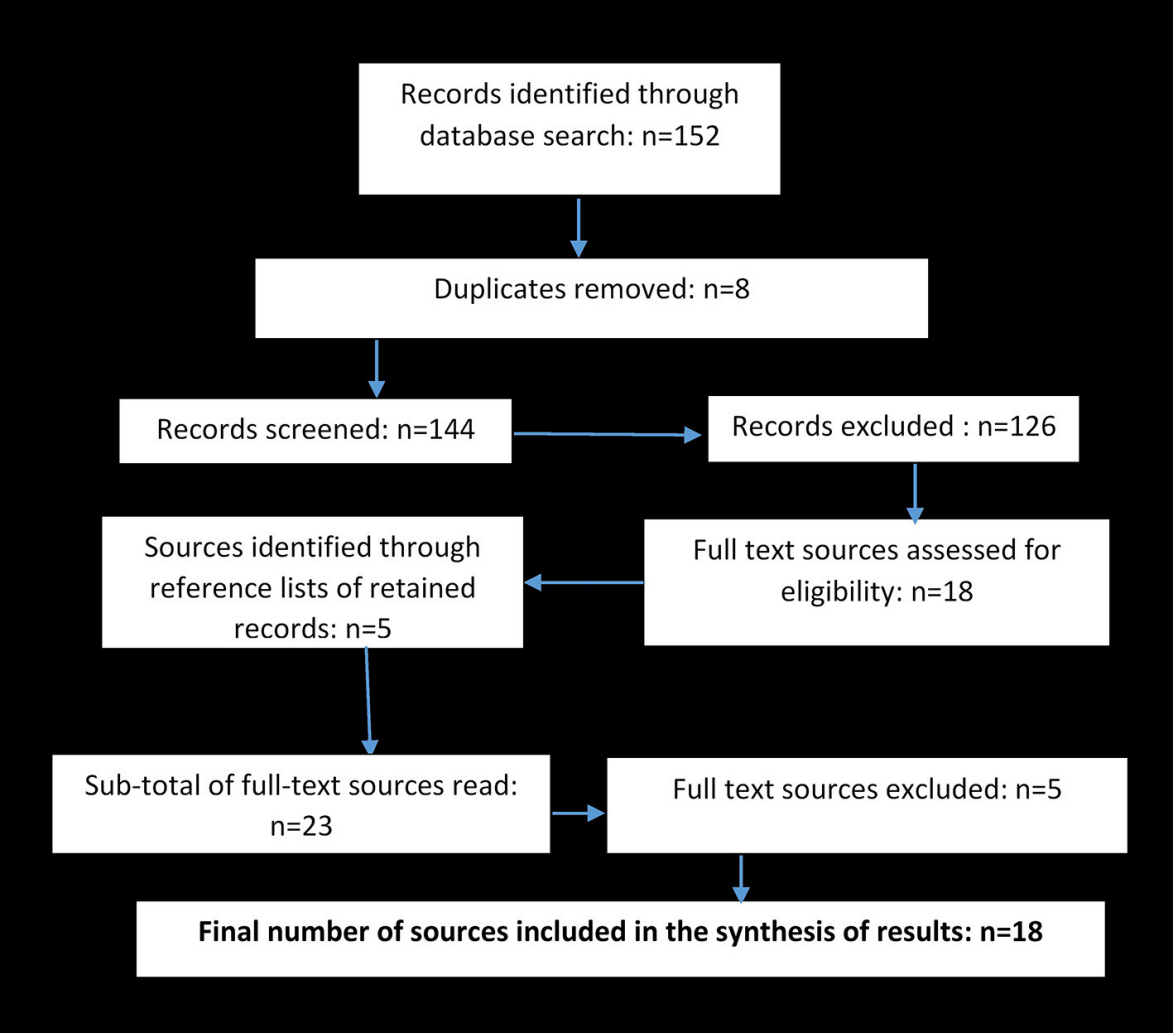
Records searched and sources retained.

Three databases were searched: ERIC, PsychInfo and Web of Science, with the Web of Science database refined to include items in the categories of music, education, gerontology, interdisciplinary social sciences, rehabilitation, and multidisciplinary psychology. Focusing on abstracts, search terms were: music, technology, aging (aging, elderly, seniors, older people, older adults). Searches were therefore carried out in the following manner: (1) music + technology + aging; (2) music + technology + aging; (3) music + technology + elderly; (4) music + technology + seniors; and so on. The criteria for inclusion were that the paper should: (1) be in English; (2) report empirical research involving the use of music technologies intended to support receptive (listening, interpreting, reflecting) or active (playing, creating, performing) engagement with music amongst older persons; (3) include research participants who may be classified as “older people,” defined as being aged 60 years or above (United Nations, 2017); (4) be published as a peer reviewed journal article. After removing duplicates, a total of 144 records were found through database searches, and their titles and abstracts were screened against the inclusion criteria. One hundred and twenty-six records were excluded at that stage, while 18 records were retained. A further five records were identified and added, through the process of scanning the reference lists of these 18 retained records (Figure 1). Full texts for the sub-total of 23 retained records were located and read in full. Five papers were excluded for reasons that included: (1) the role of music was as a stimulus rather than as a medium for musicking, according to the categories proposed by Elliott and Silverman (2015); (2) the focus was not on musicking, but on media more broadly; (3) the paper was descriptive or theoretical rather than empirical; (4) the paper was not a peer reviewed article. Thus, a final total of 18 full-texts were retained for inclusion in the review.

## Results

The 18 papers included in this review are listed in Table 1. Information extracted included study design, purpose of the study, facets of musicking addressed in the study, number and ages of participants and whether the study focused on participants living with dementia, and key findings. Overall, the majority (10) of studies focused on using technology to support musicking in the form of listening, reflecting, and interpreting. Just five studies from the total of 18 explored the utility of technology in promoting singing or playing instruments, while a further three were focused on music and movement. Ten studies focused on persons living with dementia, and of those, six focused on using technology to support listening, while three included an element of promoting movement with music and just one included making music with a digital instrument.

**Table 1:** Full text sources retained.

**Author, date**	**Study design**	**Purpose**	**Form of musicking**	**Sample size (age range in brackets)**	**Sample focuses persons with dementia**	**Key findings**
Belgrave and Keown, 2018	Mixed method Intervention study (4 weeks)	Examine changes in cross-age comfort and expectations in intergenerational virtual choral music-making.	Singing, performing, reflecting	*n* = 18 (61–79) *n* = 14 (9–14)	No	Use of simple and free computer technology supported communication, learning, and enhanced relationships between opposite generations.
Connell, 2012	Ethnography: four projects of 4 weeks each, over 3 years.	Explore the role of music and music technology in the construction of generational identity, through intergenerational workshops.	Listening, reflecting, interpreting	*n* = 48 (50–80) *n* = 29 (12–18)	No	More generational continuity in musical taste than expected. Identified main paradigmatic shift in the 1950s.
Davison et al., 2016	Randomized, single-blinded, cross-over trial over 8 weeks	Test the effect of digital Memory box (including music) on anxiety, depression, and agitated behavior.	Listening, reflecting	*n* = 11 (75–95)	Yes	Preferred music and photographs most popular. Reduction in anxiety and depression.
Duffey et al., 2008	Case study with qualitative methods	Use music as a medium for reminiscence and life review (Musical Chronology process).	Listening, reflecting, interpreting	*N* = 1 (76)	No	Reminiscence, in the form of music sharing rituals, is one way for older adults to reflect on their pasts, share memories with loved ones and productively integrate their experiences.
Ellis, 2004	Longitudinal (30 weeks) exploratory case study. Video analysis using layering approach.	Explore potential benefits of vibroacoustic therapy.	Listening, playing, creating	*n* = 2 (1 age 8, 1 mid 80s)	Yes	Improvements In mood, interaction, communication, physical expression.
Garland et al., 2007	Randomized single-blind experiment	Compare the effectiveness of simulated family presence and preferred music in reducing physically and verbally agitated behaviors.	Listening	*n* = 30 (66–93)	Yes	Even simple technologies can enrich residents' lives and alleviate distress. Preferred music was moderately successful in improving physical agitation.
Laes, 2014	Qualitative narrative study	Interrogate the aims and values of music education in the form of a rock band for older women, within a formal music education context.	Playing, performing	*n* = 6 (~70)	No	The Rock Band functioned as a context where participants explored a rock musician identity; experienced feelings of affirmation; and developed a sense of pride, achievement and success.
Lancioni et al., 2014	Within-subjects experiment	Assess basic technology-aided strategies that would allow independent choice of music.	Listening, reflecting	*n* = 4 (75–89)	Yes	Technology-aided programs allowed patients with moderate Alzheimer's disease to select their preferred music options and activate music pieces independently. Behavior during the program was also rated favorably.
Miquel-Romero and Montoro-Pons, 2017	Structured questionnaire	Analyze recorded music consumption habits, technologies of choice.	Listening	*n* = 315 (18–70)	No	Older adults preferred traditional technology formats, but engaged with new formats when these were perceived as personally relevant.
Pike, 2011	Mixed method longitudinal study	Explore the experiences of older people making music together using portable MIDI keyboard.	Listening, playing, performing, reflecting	*n* = 35 (65–95)	No	Music technology enabled the participants to play at a more musically sophisticated level than they would be capable of otherwise, and supported sustained engagement in the learning process.
Reid et al., 2017	Between-groups experimental design	Test a technology-based method for conducting longitudinal studies of regular singing and song learning in older adults.	Listening, singing	*n* = 4 (65–84)	No	Technology-based singing and song learning protocol was shown to be feasible for use with older adults
Rosseland, 2016	Qualitative research-through-design study, using observational methods	Explore interpersonal synchrony and entrainment between paired participants with Alzheimer's Disease, aided by an interactive system that synchronizes the musical tempo and the tempo of the users' movements.	Listening, movement to music	*n* = 9 (“elderly”)	Yes	Most participants were able to use the device and experienced entrainment and synchronous movement, when working in pairs.
Rosseland and Culén, 2016		Describe and discuss a Research & Development (R&D) project focused on designing a rhythmic interaction device for older people.	Listening, movement to music	*n* ≈ 12 (“older people”)	Yes	Highlights how R&D involving users can generate new knowledge. Repmoves promoted freedom of movement, and entrainment.
Scott Reis et al., 2012	Research & development project	Develop a bespoke music technology for use by the elderly that allows musical expressiveness through motion	Listening, movement to music	*n* = 8 (Mean age 83)	No	Seven out of eight succeeded in using the technology to create expressive movement and sound.
Sixsmith et al., 2007	User-led research and development	Design simple music player for persons with dementia, with user-led process.	Listening, reflecting	*n* = 26 (62–96)	Yes	Integrated theoretical, methodological, and design framework, leading to development of prototype,
Smith et al., 2017	Repeated measures within participants design	Determine if a self-administered computer-based rehabilitation program, could improve music appreciation and speech understanding in adults who have a cochlear implant.	Listening, reflecting, interpreting	*n* = 21 (32–82)	No	Significant pre-post improvements for low musical ability participants, with regards music enjoyment, aural acuity, music and speech perception.
Tak et al., 2015	Observational descriptive study	Identify characteristics that are crucial for the success of a computer-assisted intervention for persons with dementia.	Listening, reflecting	*n* = 27 (78–97)	Yes	On average, participants engaged in 3 sessions per week of 25 min each. They required an average of 2 min technical assistance per session. Listening to music was the most preferred activity.
Vahia et al., 2017	Observational study	To investigate the feasibility, safety, and utility of tablet devices in managing older psychiatric inpatients with agitation and dementia.	Listening	*n* = 36 (Mean age 80)	Yes	All participants, regardless of dementia severity, were able to use apps that included music apps, and were rated by staff to derive clinical benefit. Music apps were among the most frequently used type of App.

### Using Technology for Accessing Preferred Music

A growing body of evidence indicates that older people are capable of learning to use music technology to support access to listening and reflecting on their preferred music. For example, Lancioni et al. (2014) reported that older people living with mild to moderate Alzheimer's Disease were able to engage with a technology set-up comprising a laptop computer, amplifier, user interface (switch), and speaker intended to support the process of choosing and accessing their preferred music. Four participants aged 75–89 took part in an intervention (preceded by guided practice attempts) that comprised 10 min sessions where they were invited to use the technology to access their music choices. Prior to this experimental intervention, control baseline sessions took place where staff played recordings for the participants, without the aid of the technology set-up. Video recordings of the baseline control sessions and the experimental intervention sessions were evaluated using a social validation check that included indicators of happiness, self-determination, and social image. Results revealed that participants were able to use the technology, and the social validation scale differentiated between the control and experimental (technology) sessions, with more positive scores found in the experimental sessions where participants used the technology.

The idea of using simple technologies as an interface for enabling access to preferred music was also explored by Davison et al. (2016). In their within-subjects experimental study, 11 participants aged 76–95 and living with dementia completed a protocol that involved learning to use a Memory Box–a laptop computer configured in such a way as to provide easy access to personally meaningful music, photographs, movies, or messages. Over 8 weeks, each participant spent a total of two and a half hours learning to use the Memory Box with researcher support (the experimental intervention), and an equivalent amount of time in visits from a researcher who read the newspaper and discussed local events (the control condition). Measures for anxiety, agitation, and depression were completed pre and post intervention. Overall, participants showed a preference for music and photographs, and several (six out of 11) were able to use the system independently. Anxiety and depression were both found to decrease, over the period of the intervention. These findings added to an earlier study by Garland et al. (2007), who carried out a within-subjects experimental study with older people living with dementia. Thirty participants were exposed to a music listening condition, which was compared to a simulated visit (audio tape of a familiar person), a placebo (audio tape of a gardening book), and no intervention. Observations were made, using a time sampling approach before, during, and following each of the conditions. Listening to personally meaningful music was found to have moderate and variable positive effects for physical agitation. A key finding here was that a simple technology (in this study, a cassette player and headphones, with a playlist comprising personally significant music) could function as a tool to alleviate physically aggressive behavior among older persons with dementia.

In a similar vein, Sixsmith et al. (2007) demonstrated that older persons with a dementia diagnosis expressed a desire to use technology and were able to do so in order to access preferred music. Their observational study aimed to understand the factors that could best support older people with dementia in engaging with computer activities. Twenty-seven nursing home residents with an average age of 85 were offered the opportunity to take part in daily computer activities (including a range of choices that included music, games, video, email, and internet searching) over the course of 7 weeks. On average, participants voluntarily attended three sessions per week of approximately 25 min each. Participants engaged successfully with an average of two activities per session, requiring just 2 min (on average) of technical support during each session. The researchers noted that the most liked activity was listening to personally meaningful music that accompanied slideshows or videos.

With a similar focus on using technology to enable autonomous creative choices and access to preferred music, Sixsmith et al. (2007) describe a user-led research and development initiative in the UK, focusing on the creation of a simple music-playing device that would meet the user needs of older people. An ecological model of quality of life, taking account of personal attributes (e.g., cognitive and psychological characteristics, preferences), contextual factors (e.g. social support, physical characteristics of the immediate environment), and socio-cultural factors, framed an iterative process that led to the development of a device intended for use by individuals living with dementia. This ecological model “focused on the everyday activities of the person and highlighted opportunities for targeting technology and design solutions” (p. 87). Through initial interviews with 26 participants living with dementia and subsequent stakeholder focus groups, both music and creativity were identified as key themes related to quality of life. “Music was important … Music provided a context for a variety of forms of interaction and participation and had beneficial emotional effects” (p. 89), and “promoting the enjoyment and use of music” (p. 90) emerged as a priority area on the technology wish list. A prototype was developed, involving input from a range of perspectives (e.g., engineers, designers, gerontologists, dementia researchers), including testing and feedback from end-users (persons living with dementia). Sixsmith et al. (p. 92) explain that “this ensured that technology development took place in a way that was based on the experiences and needs of people with dementia and their caregivers rather than being based on a technology-driven approach.”

Focusing on accessibility and the potential wider benefits of technology, Vahia et al. (2017) investigated the use of tablets as a non-pharmacologic intervention intended to reduce anxiety amongst patients at a geriatric behavior health inpatient unit. Two iPads were each loaded with 70 applications (apps), including music and musical instruments as well as communication, games, video entertainment, news, and photography apps, rated for level of complexity. When patients became agitated or restless, they were given an iPad and directed toward apps that matched their individual preferences. Music playing apps, and particularly harp and piano, were amongst the five most commonly used. Time spent engaged with apps, the specific apps chosen, and a rating of restlessness after app use were recorded. The authors reported that all of their 39 participants tolerated the use of the tablets, although time spent using the app as well as the level of app complexity were negatively related to severity of dementia. Those diagnosed with mild dementia benefitted the most with regards to reduction in agitation.

The specific technologies of choice for accessing music, among community-dwelling adults, have also been explored. Miquel-Romero and Montoro-Pons (2017) carried out a structured questionnaire study, using a quota sampling method to achieve a representative sample of 315 Spanish residents aged 18–70, 34% of whom were aged 46–70. The questionnaire gathered information about music listening habits including devices used, opinions about music, and socioeconomic data. The researchers found that overall older people were not enthusiastic about accessing music using new digital formats, preferring to rely on hi-fi systems, DVDs, or the television, suggesting that the music industry must recognize the continuing need for supporting these formats. However, the authors also point out individual differences, and highlight that older adults are only likely to engage with new technological interfaces when these are perceived as being personally relevant and meaningful.

Overall, the research concerned with using technology to enable access to preferred music in later life contexts demonstrates that technologies can mitigate barriers to personally meaningful musicking in the form of listening and reflecting. The key messages are that the technology must be relevant to the needs of the user, and that “under caregiver supervision, even persons with severe impairment may use simple and intuitive apps, especially when they are matched to each individual's preferences and level of cognitive function (Vahia et al., 2017, p. 863).

### Using Music Technology for Reminiscence

The significance of music technology for supporting reminiscence has been highlighted. For example, Lazar et al. (2014) carried out a systematic review of technology as an aid for reminiscence therapy and found that eight out of ten studies that described using multi-sensory reminiscence approaches had integrated recorded music alongside other stimuli. Reminiscence therapists used technology, in these instances, as a tool to harness musical responsiveness, described as a continuing ability that persists long after other abilities may have become compromised.

Music technology as a specific tool for reminiscence and life-review was investigated by Duffey et al. (2008), who developed a Musical Chronology process, a structured approach whereby clients recollect and share popular songs that are accessed using technology and mapped against significant points in their life journeys. The Musical Chronology has been applied with older adults where there have been reports of “multiple areas of personal growth, including increased self-awareness and self-acceptance, the development of new relationships, increased comfort with being open and authentic in relationships, progress in dealing with grief, a renewed appreciation for life, and hope for the future” [Somody, 2010, p. v–vi]. Duffey et al. (2008) describe the adaptation of the Musical Chronology as a framework for collaborative life review between adult children and their parents, using one case study to demonstrate how music sharing rituals, supported by technology, offered the opportunity “for older adults to reflect on their pasts, share memories with loved ones, and productively integrate their experience” (p. 60), the latter point referring to a major later-life developmental task (Erikson and Erikson, 1998).

Intergenerational musical review was also investigated by Connell (2012), who explored the role of music and music technology in the construction of generational identity. Over 4 years, four community projects took place, involving a total of 48 older people aged 50+ to 80+, alongside a total of 29 young people aged between 12 and 18. The participants were trained in the use of DJ equipment, and given access to this equipment as well as a supply of vinyl records, over a series of four worksops. The workshops were found to provide a powerful context for enhancing intergenerational understandings. Overall, although the 1950s was identified as a period where there had been a paradigmatic shift in musical identities, the workshop activities revealed more generational accord than discord. As Connell reports, “one of the more striking findings so far is that actually, for older people aged 70-something or less, many of their tastes are not so far away from those of teenagers as initial stereotyping might suggest” (p. 275).

In these examples, it is clear that the technology becomes a means to an end, functioning as the tool with which users may explore their unique lifecourse narratives. Technology may also serve as a tool for using music as the focus of intergenerational dialogue, enhancing relationships and reciprocal understandings of generational values and identities.

### Music Technology to Support Singing

Significant wider benefits of singing in later life have been demonstrated (Clift et al., 2008), but there has been limited research focused on ways in which technology can be harnessed to support older people in engaging with singing. Reid et al. (2017) adapted a commercially available iPad application (SingFit) and tested its efficacy as a technology-based method for conducting longitudinal studies of regular singing and song learning in older adults. Forty-eight musically inactive participants (i.e., they had not engaged actively with music-making for at least 40 years) aged 65–84 were allocated to singing, listening, or control groups. The singing group used the app as a “sing-along” aid to learning and re-learning to sing favorite songs. The listening group used the app as a listening device, also focused on their favorite songs, while the control group did not use the app. The researchers were interested in the feasibility of using the app to promote singing, and whether daily singing or listening over a period of 5 weeks could have a positive effect on cognitive function. Although no significant effect was found for cognitive function (possibly because the 5 week protocol was too short to reveal any such effects), a key finding of this study was that the app functioned as an accessible gateway to singing. Older adults used the app with minimal support, with the majority of participants finding it motivating and enjoyable some reporting that they intended to continue singing after the research protocol had ended.

### Technology to Support Music Perception and Appreciation

It is known that hearing loss is widespread among older people, and that there are significant challenges associated with this (Lin et al., 2011). One such challenge may be the loss of music perception, and consequently diminished enjoyment of musicking, in any of its forms. Accordingly, some researchers have focused on the potential for technology to serve as a tool to support rehabilitation in relation to music perception, among adults with cochlear implants (Smith et al., 2017).

Smith et al. (2017) recruited 21 adults aged 32–82 and living with a cochlear implant, who participated in a study to determine if a self-administered computer-based rehabilitation program, HearTunes (Rehab), could improve their music appreciation and speech understanding. The software presents musical patterns structured in such a way as to incrementally improve listening and focus. A novel feature of this software was its holistic nature, encompassing all aspects of musical perception rather than focusing solely on isolated musical tasks. Participants, who were categorized as having high or low musical ability (according to data gathered regarding musical background), engaged with the software for an average of 3.5 h per week, over 4 weeks. Pre and post tests were carried out, measuring music enjoyment, music perception, and speech understanding; the tests were repeated again 6 months after the study had finished. Significant improvements in enjoyment of music were found after 4 weeks, for the low-ability music group. At the 6 month follow-up, improvements in enjoyment of music were also found for those designated as having high musical ability. With regards to the tests for aural acuity, perceptual ability, and speech recognition, the software seemed to be most effective for those in the low musical ability group, although the significant improvements from pre to post training were not always evident at the 6 month follow-up. The researchers suggest that a longer rehabilitation period, initiated closer to the activation of the cochlear implant (CI), may be necessary in order to achieve long-lasting results, and reflect that “more complex and challenging versions of the software may also be necessary to develop to elicit more significant improvements in those CI users with more experience and background in music” (p. e267). Overall, a key finding of the study is that technology can be mobilized for supporting older adults in regaining and sustaining the capacity to engage with music, notwithstanding hearing loss. However, once again this study demonstrates the need for technology to differentiate for specific user needs and characteristics.

### Collaborative Musicking Supported by Technology

Very few studies have focused on the potential for later-life active and collaborative musicking supported by music technology. However, technology as a vehicle for group music making was investigated by Pike (2011), who explored the experiences of older people making music together using portable MIDI keyboards connected through a mixer, with the cohesive group sound broadcast through portable speakers. Thirty-five older people aged between 65 and 95 participated in the group over the course of 8 years. Data were collected from the participants via questionnaires, interviews, and recorded observation. While none of the participants had previous experience of using MIDI keyboards, this did not pose a barrier to learning but rather enhanced the overall musical experience. Specifically, the music technology was perceived to have enabled the participants to play at a more musically sophisticated level than they would be capable of otherwise, and supported sustained engagement in the learning process. Participants highlighted that the MIDI technology enabled participants to hear their individual parts within the group, served as an important tool for supporting the group in developing an internalized sense of pulse, and that the option to gain immediate aural feedback promoted discussion focused around creative choices relating to timbre, orchestration, and articulation.

The implications for music education values and priorities were explored by Laes (2014), who carried out narrative interviews with older women who, as musical novices, participated in a rock band within a formal music education context. As Laes (p. 11) explains, “ music pedagogues of today are challenged to have a responsibility to engage third-age learners with music learning, for example by exploring the use of technology and the design of collaborative learning environments, and both these goals are easily realized in a rock band context for third-age learners.” The key themes emerging from Laes' study were that the rock band was a context for learning and participation where the participants had the opportunity to explore their musical identities, and where they experienced a sense of social affirmation, pride and success, as successive challenges were overcome.

Intergenerational asynchronous collaboration using an online platform was the focus of a study reported by Belgrave and Keown (2018). In their research, an established music therapy choir comprising older adults aged 61–79 collaborated in a project with a youth choir, aged 9–14. The groups engaged in two “virtual exchanges,” in the form of recordings of themselves shared via Dropbox. The first of these recorded exchanges consisted of an introduction to their choir, followed by a recorded performance of a piece of music that was deemed to be representative of the choir. The second exchange comprised a teaching demonstration, whereby each choir provided an audio recording of themselves teaching a favorite song to their counterparts. Following the virtual exchanges, the two choirs met in a face-to-face workshop and performance. The participants filled in pre and post measure of intergenerational attitudes and expectations of the intergenerational experience. A key finding of the project was that the use of a simple and free computer technology supported communication, learning, and enhanced relationships between the opposite generations. As the authors noted, “both generations reacted positively to the interactions and cited the [virtual] interactions as one of the top enjoyable factors of the project” (p. 7).

The reported research concerned with using technology to support collaborative playing and performing is scarce. However, there seems to be some indications that even simple technologies can indeed support musical learning amongst older people, that older adults happily engage with musical activities involving technology, and that there may be important potential wider benefits associated with the use of music technology in intergenerational contexts.

### Music and Movement With Creative Music Technologies

One interactive and creative music technology that has perhaps been under-researched (although used widely in special needs contexts) is the Soundbeam, described as an “elastic keyboard in space that allows sound to be created without the need for physical contact with any equipment” [(Swingler, 1998), p. 2]. The Soundbeam works on the same principle as the Thereminvox, in that high frequency ultrasound beams, inaudible to the human ear, are emitted and movements within those beams are transformed into musical sounds. However, in contrast to the Thereminvox, the Soundbeam, which translates distance and movement data into a digital code that is interpreted by any electronic instrument or sound, offers infinite possibilities for the quality of soundscape that is created. A second key difference that distinguishes the Soundbeam from the Thereminvox is that the beams emitted by Soundbeam can be adjusted to lengths varying from a few centimeters to several meters, making it possible to control musical sound with movements ranging from very small to very big. In addition, the sounds created can be transposed, making it possible to integrate the Soundbeam within collaborative music-making involving acoustic instruments. Finally, the Soundbeam offers scope for adjusting the number and sequence of notes captured within each beam (e.g., a particular scale with a specific number of octaves), to define the articulation required to trigger sound, and to adjust specific qualities of the sound produced. Thus, individuals with varying degrees of mobility are enabled to participate in intentional and collaborative musical activities, with the scope for making aesthetic choices, expressing imaginative ideas, exercising choice, developing listening skills, enhancing confidence, developing spatial awareness, and refining motor skills (Russell, 1996; Swingler and Brockhouse, 2009).

The Soundbeam has been found to be “the single most frequently used electronic music technology” by UK music therapists (Magee and Burland, 2008, p. 125). A survey of 22 special educational needs schools in the UK revealed that 30% of music therapists within those contexts had used music technology, and of those 30, 76% had used the Soundbeam in their practice (Farrimond et al., 2011). However, the overall use of assistive technology in music therapy is not yet widespread, and much of the published evidence in that domain is concerned with the use of electronic music technologies with children or adolescents (Magee, 2013). For example, Hahna et al. (2012) carried out a survey of technology use within music therapy contexts in Australia, Canada, the UK and the USA. Overall, 71% of survey respondents reported using music technology in their clinical practice, but of those just 16% used technology with clients over the age of 65.

A notable exception is Ellis (2004), who reports a longitudinal case study example of using digital music technologies with an elderly resident (aged mid-80s) within a long-term care context, who had been languishing following a stroke. Aiming to improve communication, motor control, and well-being, Ellis had developed the Vibroacoustic Sound Therapy (VAST), comprising exploration with a microphone and sound processor (supporting enhanced interactive communication skills), music-making with the Soundbeam (supporting independent physical movement and control, extending listening range, awakening curiosity, enabling self-expression), and dedicated time relaxing in the “Soundchair,” where recorded low-frequency calming music is intended to promote physical and mental well-being and to potentially trigger memories and reminiscence.

Ellis (2004) describes a layered approach to analysis of video recordings of several sessions over time, where VAST was applied. In the layered approach, significant moments from each session are extracted and layered one after another, creating a chronological representation of progression concerning specific behaviors or responses. Engaging with the Soundbeam, the participant “spontaneously started to use her left arm to control sound in most expressive ways,” and after several weeks she was reported to be generally more calm, communicative, cheerful, and sociable. These findings are framed by a summary of field notes gathered over several years, documenting the effects of VAST for over 35 individuals in later-life therapeutic contexts., where Ellis reports having observed improved communication, mobility, opportunities for individual exploration and control, deep relaxation, pleasure, well-being, and self-esteem. The importance of facilitation strategies is highlighted by Ellis, who cautions that technology must be mobilized in such a way as to support intrinsic motivation and a locus of control in the user.

With a similar focus on using technology to promote interaction with sound, a more recent Research through Design project, whereby prototypes are developed through a reflective process involving user engagement in the field, addressed the question of whether a music player could inspire seniors to be more physically active (Rosseland, 2016; Rosseland and Culén, 2016). The researchers describe the development of RepMoves, articulating “a generic interaction design concept to motivate physical activity through rhythmic interaction with music” (Rosseland and Culén, 2016, p. 108). Using a motion sensor, the RepMoves prototypes adjusted the tempo of its music to the tempo of five specific movement patterns performed by the user. Essentially, the user's role is conceptualized rather like an orchestral conductor, with his or her body movements such as arm swings or body sways dictating the tempo of the “orchestra” (in this case, the music player RepMoves). RepMoves prototypes were tested with groups of older people diagnosed with early stage Alzheimers Disease. Through exploratory sessions using familiar songs, collaborative movement with RepMoves was found to be possible, stimulating entrainment, or moving in synchrony with one another, a phenomenon that has been associated with positive well-being (Gill, 2012). Visual feedback in the form of photographs and videos was also added, offering an experience close to a virtual reality. The researchers illustrate that, through the conceptual exploration with older people, they “were able to explore and understand how a range of contextual, personal and social factors could influence the future adoption of the RepMoves concept in specific contexts” (Rosseland and Culén, 2016, p. 116).

The idea of using motion sensor technology was likewise explored by Scott Reis et al. (2012). Their research and development project was framed by the underpinning principles of accessibility, inclusion and social participation. The aim was to develop a bespoke music technology for use by the elderly that would “allow musical expressiveness through motion, solely using the resources available in an ordinary home computer” (2012, p. 211). A prototype was developed that recognized motion (using a computer camera), and translated it to an audio signal that was then reproduced as audio, with low latency. The prototype was tested with a group of eight older people with an average age of 83, none of whom had background experience with music-making or with using computers. The participants were curious, excited and happy to explore the new technology, and seven out of the eight participants succeeded in making expressive musical sounds within 10 min trials.

Overall, the limited research that has thus far addressed the question of how technology can encourage expressive movement with music points to strong potential within gerontechnology for the development of music technologies that support enhanced mobility, expressivity through music, with implications for positive well-being.

## Discussion

This review of literature has demonstrated that the design and use of creative music technologies intended to enrich later life remains an under-researched area. Much of the literature that does exist focuses on music technology as an assistive tool within therapeutic contexts, while there is very little research, to date, that explores the affordances or challenges relating to engagement with music technologies amongst older people living independently within the community. Furthermore, there is limited research concerned with what inclusive practices using music technologies in the community, with intergenerational or later-life groups, might entail. However, this review does synthesize a growing body of research, design and practice that has recognized the significance of music as a continuing ability that may be supported in personally meaningful, creative and differentiated ways with accessible music technologies (Lazar et al., 2014).

The literature reviewed in this paper suggests that older people, even those with complex needs, are capable of engaging with technology and using technology in a range of ways that support their musical perception, learning and participation (e.g., Pike, 2011; Laes, 2014; Smith et al., 2017). This evidence is in accordance with research concerned with later-life musical engagement more generally, where it has been demonstrated that older learners develop compensatory strategies to mitigate physical or cognitive constraints (Gembris, 2008). Technologies that support receptive musicking (e.g., listening devices) as well as active musicking (e.g., motion sensor devices, digital musical instruments, singing apps and music composition or improvisation technologies) have been found to support access to the multiple personal, social, cognitive, and physical benefits that have been associated with musicking (e.g., Ellis, 2004; Rosseland, 2016; Reid et al., 2017; Vahia et al., 2017). Furthermore, music technologies have been shown to function as creative tools that may have the capacity to provide “restorative spaces” (Jansen, 2005) that privilege reflection and reminiscence, personal healing and problem solving (Duffey et al., 2008; Somody, 2010; Connell, 2012; Lazar et al., 2014). These activities may be of particular importance at a stage of life where the potential for creative growth and expression (Erikson and Erikson, 1998; Weisberger, 2013) may be overshadowed by narratives of decline (Findsen, 2005). Thus, the creative use of music technologies, as evidenced in this literature review, offer strong potential to serve as a vehicle whereby older people may overcome barriers to musicking and, through music, make substantive connections in their lives (Duffey et al., 2008) and engage in purposeful and enriching activities.

What then are the underpinning theoretical principles that could be said to frame the design of creative music technologies that enrich the lives of older people? The literature reviewed here demonstrated that that effective and innovative design of music technologies for older adults requires cognizance of the diversity amongst the intended audience. Indeed, it has been argued that as people accumulate life experience they become more different than similar, and that attempts to characterize older people as a homogeneous group thus risks being /misguided and ageist” (Withnall, 2010, p. 119). The evidence that diverse groups of older people can and do engage with music technologies may thus encourage designers to reflect upon their own underlying assumptions about older people and to engage with research and development approaches that place the users at the heart of a design process with a strong element of user testing and feedback in the field (Sixsmith et al., 2007). Approaches to the design of later-life music technologies may be particularly salient. When they are: (1) inclusive in embracing an ecological model of quality of life (Sixsmith et al., 2007) that recognizes the prior experience and wider socio-economic and cultural characteristics of participants' lives; (2) accessible, in facilitating engagement and allowing older adults to recognize and implement compensatory user strategies; (3) are assistive, in enabling interdependent creative expression, learning and participation, and (4) universal, in the sense that the technologies function as a space for the collaborative and social practice of musicking.

Some limitations to this review relate to the rapid advances in technology itself. As the review was limited to published peer-reviewed journal articles, some cutting-edge innovations in the use of music technology in later-life contexts, not yet the focus of published empirical user-studies, may have been omitted. Indeed, excluded records included, for example, conference proceedings that reported the design and development of piano-playing robots intended to foster positive affective states among older people (Park et al., 2008), computer software using motion technology to foster musical expressiveness (Reis et al., 2012), as well as mobile Apps intended to promote easy access to personally meaningful music (Nezerwa et al., 2014; Wang and Tan, 2015). Notwithstanding their non-inclusion in the systematic review reported here, these sources reinforce the key finding that older people can and do engage with technology, and that the development of technologies that facilitate (1) access to preferred music and (2) the use of music to reduce stress, particularly among persons living with dementia, has been of particular interest. A further limitation is language. This systematic review represents peer reviewed studies published in English; yet our aging population is a global issue of concern to researchers worldwide working in the interdisciplinary fields of gerontechnology and music, health, and well-being.

In conclusion, notwithstanding the limitations noted above, this review provides a strong rationale for exploring and developing the landscape comprising music technologies for older people in a range of diverse contexts. Arguments focused around quality of life, cognitive function, social interaction, and supporting mobility all point to the need for research and development in this area. Perhaps the strongest argument of all though is the view that innovative music technologies may enable access to joyful, creative, and restorative experience, throughout our later lives.

## Author Contributions

The author confirms being the sole contributor of this work and has approved it for publication.

### Conflict of Interest Statement

The author declares that the research was conducted in the absence of any commercial or financial relationships that could be construed as a potential conflict of interest.
